# Multimodal Therapeutic Approach in Women with High Risk of Metabolic Syndrome—A Single Group One Center Pre-Post Study

**DOI:** 10.3390/jcm10214915

**Published:** 2021-10-24

**Authors:** Jagoda Rusowicz, Anna Serweta, Wojciech Idzikowski, Joanna Szczepańska-Gieracha

**Affiliations:** 1 Department of Physiotherapy, Wroclaw University of Health and Sport Sciences, 51-612 Wrocław, Poland; joanna.szczepanska@awf.wroc.pl; 2 Department of Physical Education and Sport Sciences, Wroclaw University of Health and Sport Sciences, 51-612 Wrocław, Poland; serwetanna@gmail.com (A.S.); wojciech.idzikowski@awf.wroc.pl (W.I.)

**Keywords:** postmenopausal age, stress, mood, mental health, obesity, public health

## Abstract

The study aims to determine the impact of multimodal therapeutic approach on self-perceived stress in women with high risk of Metabolic Syndrome (MetS). The study involved 43 women aged 60 years and over (mean 68.6 ± 6.5) participating in a Mental Health Promotion Program. Over the 3-month course of the project, all of the participants attended meetings of a support group (60-min sessions twice a week). During these meetings, they took part in general fitness training (20 min), dancing (20 min), as well as health-promoting education and psychoeducation sessions (20 min). Moreover, the participants were encouraged to modify their diet to reduce their daily fat and sugar intake. Stress levels were assessed using the Perception of Stress Questionnaire (PSQ). Mood was measured with the Geriatric Depression Scale (GDS-30). In all of the subjects, a body composition analysis was performed using a Tanita BC-545N analyzer. Abdomen and hip circumference were measured to determine the waist-hip ratio. Weight and height were measured to determine the BMI score. At the beginning of the project, the intensity of stress correlated with the level of depressive symptoms (GDS), Body Mass Index (BDI), and the amount of visceral fat. Three months of participation in the Mental Health Promotion Program resulted in a significant reduction in stress intensity (*p* < 0.01). At the end of the project, all of the participants expressed their willingness to continue their participation in the classes, which is very important as there is a need to conduct long-term health-promoting activities in the age group in question.

## 1. Introduction

From the years 2015 to 2050, it is expected that the percentage of the world’s population aged 60 or more will increase from 12% to 22% [1]. The world is facing the challenge of developing health and social care systems that will be prepared for these demographic changes. Its consequence will be a general increase in the incidence of cardiovascular disease, obesity, cancer, and other health issues typical for older age. Most of the elderly population are women (58%), as there are 140 women per every 100 men [2,3]. Therefore, we must seek effective, universally applicable, and cost-effective programs for health promotion and disease prevention in the elderly population.

In the above context, given the complex mutual dependencies between health, physical fitness, and psychosocial problems, a biopsychosocial approach deserves particular attention. In this approach, great emphasis is placed on the group nature of all the activities (reducing the sense of loneliness and social isolation), on body training (general fitness training, relaxation, dance), as well as health promotion and psychoeducation. This holistic therapeutic approach must be carried out on a long-term basis by a multidisciplinary team of specialists (e.g., a physiotherapist, a psychotherapist, and a music therapist) experienced in working with older patients [4]. The biopsychosocial model can be used to improve clinical outcomes by building patients’ awareness of the interactions between biological, psychological, socio-cultural factors, and to increase patients’ self-management of illness [5].

The postmenopausal period is the time of the higher risk of mood disorders. Although not every person experiencing high-stress levels suffers from depression, these people are at high risk of developing depressive symptoms. Stress is a set of reactions the body responds with to stressors that place its coping abilities to test. The main causes of stress are change and the need to adapt to the biological, social, physical, and environmental requirements. The life changes experienced by the elderly become stressors that have a detrimental effect on their health and functioning. The postmenopausal period in women may have a significant role in the occurrence of adaptive disorders leading to high levels of stress. A decrease in estrogen production results in its reduced protective activity in the circulatory system, which, in consequence, increases the risk of cardiovascular diseases [6].

Our previous study demonstrated that low-intensity physical exercise used in a group setting combined with psychoeducation resulted in significant improvements in both depression and stress levels in women with MetS [7]. Dance intervention may be an effective adjunct therapy to improve mood and physical function in adults [8]. Moreover, it may be a relevant form of exercise for older adults due to social factors and accessibility [9]. Furthermore, dance, regardless of its style, can significantly improve muscular strength and endurance, balance, and other aspects of functional fitness in older adults [10]. As a result, we decided to include dance as an important element of our therapeutic program.

The study aims to determine the impact of multimodal therapeutic approach on self-perceived stress in women with high risk of Metabolic Syndrome (MetS), and to analyze the relationship between the level of perceived stress, mood, and the components of the MetS in a group of examined women.

## 2. Materials and Methods

### 2.1. Design of the Study

The study was identified as a single group one center study with pre-post design. The study was conducted in a group of 43 women aged 60 years and over (mean 68.6 ± 6.5), participating in the Mental Health Promotion Program at the Foundation for Senior Citizen Activation SIWY DYM in Wroclaw, Poland. The participants received a referral to the therapeutic program from their primary care physician due to a high risk of MetS. All of the patients received permission to participate in moderate intensity physical training. 

**Figure 1:**
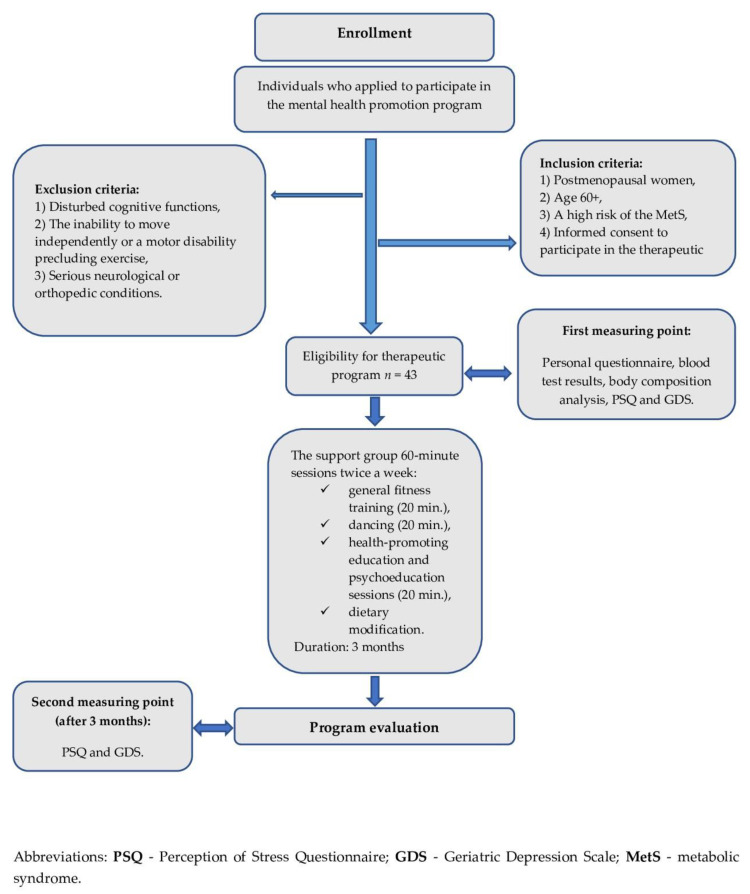
Flow chart of the recruitment process towards the study group.

Both the study and the therapeutic program lasted 3 months (12 weeks). Two measurement points were established. The first outcome measurement was taken immediately before the therapeutic program began and it included the assessment of the stress level and mood. The second measurement was conducted 3 months after the start of the study, at the end of the therapeutic intervention (Figure 1). The measurements and analyses were performed by a psychologist experienced in this area. 

Body composition and anthropometric measurements (body height and weight, waist and hip ratio), as well as blood pressure and laboratory tests (e.g., blood results, fasting sugar levels) were ordered by a primary care physician only once, at the time of project recruitment.

The project received funding from the Municipality of Wroclaw. Approval to conduct the study was obtained from the Bioethics Committee of the Wroclaw University of Health and Sport Sciences in Wroclaw (No. 16.06.14). Informed consent was obtained from all of the subjects involved in the project. The study was conducted in accordance with the Helsinki Declaration. 

### 2.2. Inclusion Criteria for the Research Project

A high risk of the MetS was diagnosed using the criteria recommended by the International Diabetes Federation (IDF): Central obesity (defined as waist circumference ≥ 80 cm in females), raised triglycerides (>150 mg/dL), reduced HDL cholesterol (50 mg/dL in females), elevated blood pressure (BP; systolic BP > 130 or diastolic BP > 85 mm Hg), and increased fasting plasma glucose >100 mg/dL [11,12]. The presence of two of the five characteristics listed above qualified subjects for the project. The exclusion criteria included disturbed cognitive functions, the inability to move independently or a motor disability precluding exercise, serious neurological or orthopedic conditions (e.g., advanced Parkinson’s disease, severe stroke consequences). The classification of normal blood pressure and hypertension is in accordance with the European guidelines ESH/ESC 2018 and is based on the limit value RR 140/90 mm Hg with a division into three steps (optimal < 120/80 mm Hg; normal up to 129/84 mm Hg; high normal up to 139/89 mm Hg) and distinction of the Insulated Subtype Systolic Hypertension (ISH) [13,14]. Patients were recruited to the project by their primary care physicians based on the results of medical tests and anthropometric measurements. We do not have information on the number of people who had been screened but did not meet the inclusion criteria for the project.

### 2.3. Measurements

The Perception of Stress Questionnaire (PSQ) by Plopa (2010) was used to assess the stress levels. The questionnaire is designed to measure the structure of stress experiences. The questionnaire allows the calculation of a general score, which indicates the generalized stress level, as well as three results referring to the following dimensions: Emotional tension, external stress, and intrapsychic stress. The questionnaire comprises 27 statements and the respondent determines the degree to which a given statement concerns him or her, using a five-level Likert scale ranging from “True” to “False”. In each of the areas (emotional tension, external stress, intrapsychic stress), there are seven statements, indicating that in each area the respondent can score between 7 and 35 points. Then, all of the points are summed up to calculate an overall score, and the score can range between 21 and 105 points. The remaining six statements in the questionnaire relate to the lying scale (from 6 to 30 points). The raw score is converted into a STEN score for gender and age, respectively. A score of 7–10 STEN indicates a feeling of increased nervousness, anxiety, and problems with relaxation. A score of 5–6 indicates average intensity of emotional tension. A score of 1–4 indicates no emotional strain. The results of the individual stress components are interpreted in a similar way. The internal consistency rates for the three scales (dimensions) in the examination of adults were between 0.70 and 0.81. The factor validity of the PSQ was confirmed [15].

Mood was assessed using the Geriatric Depression Scale (GDS), developed by Yesavage et al. (1988) as a screening instrument to evaluate the intensity of depression in older adults [16]. It consists of 30 short questions with two possible answers (“Yes” or “No”). A score between 0–9 points indicates normal mood, a score between 10–19 points indicates mild depression, and a score greater than 19 points represents a severe form of depression. The reliability of the GDS was estimated using the Cronbach α coefficient and the Spearman-Brown split-half reliability formula. Cronbach’s alpha reliability coefficient was α = 0.94, and an identical coefficient value (r = 0.94) was obtained in the split-half reliability measurement of this instrument. The sensitivity and specificity of the GDS were found to be 84% and 95%, respectively [17,18].

In all of the subjects, a body composition analysis was performed using a Tanita BC-545N analyzer. Abdomen and hip circumference were measured to determine the waist-hip ratio. Weight and height were measured to determine the BMI score. All of these tests, as well as blood pressure measurement and blood analysis, were ordered by a physician and performed in a medical setting.

### 2.4. Intervention

Throughout the therapeutic program, all of the participants attended meetings of the support group (60-min sessions twice a week). During these meetings, they took part in a general fitness training (20 min) run by a physiotherapist, a dance session (20 min) run by a music therapist, as well as health-promoting education and psychoeducation sessions (20 min) run by a psychotherapist. Each class was conducted in a small, fixed group of 10–12 women. Participants were divided into smaller groups at random. General fitness exercises were aimed at increasing muscle strength and improving the range of motion in large joints. A single exercise session lasted 20 min and consisted of 22 low-intensity, general fitness exercises. Thirteen exercises were done sitting down, seven standing, and two in the hand-and-knees position. The exercises were classified as either aerobic, musculo-articular or stabilizing. The aerobic portion served as a general warm-up for the subsequent exercises, and the musculo-articular section focused on strengthening muscles and enhancing the mobility of joints in the upper limbs, lower limbs, and the torso. The stabilizing exercises were designed to improve the stability of the body and to augment spatio-visual coordination. Moreover, the intensity of exercise was low and was intended as a warm-up for the subsequent dance session.

To ensure safety, the fitness exercises and partly the dance sessions were carried out in a sitting position on specially adapted stable chairs. In the initial part of the session, the music was characterized by a slow tempo, a peaceful character, and a stable rhythmic layer. The session used Cuban music and basic salsa steps. The pace was adjusted to the participants’ abilities. When the dancing became tiresome, the participants ended the session in a sitting position. At the peak of the training session, the exercise intensity reached a moderate level. However, each person was informed that the intensity of the exercises could not exceed 14 points on the 20-items Borg Scale (an effort described as “somewhat hard”) [19]. The load lasted no more than 1 min and was introduced gradually from the beginning of the project. Each participant could stop the exercises at any time and rest until the level of fatigue decreased to a moderate or low level. 

All of the support group meetings were developed by a therapeutic team—a psychotherapist, a physiotherapist, and a music therapist. As a part of the educational activities, participants were encouraged to modify their diets: To reduce white flour and sugar foods and increase the consumption of healthy oils and vegetables in their daily menu. The presence of a psychotherapist during all of the classes was intended to accelerate and strengthen the relationship-building processes within the group, as well as to model and reinforce attitudes of openness, cooperation, and mutual support in achieving common goals. 

### 2.5. Statistical Methods

The statistical analysis was performed using the STATISTICA 13.3 software by TIBCO Software Inc. (StatSoft Polska, Kraków, Poland). The statistical significance threshold was set at *p* < 0.05. The statistical description of the data included, in the case of characteristics with continuous distributions: Determination of the mean, standard deviation, and variability range (the minimum and maximum values in an empirical distribution). Moreover, distributions of continuous characteristics were presented as the distribution series. The normality of the distribution of the continuous characteristics was determined using the Shapiro–Wilk test. The null hypothesis on the normality of distribution was rejected in the case of most components of stress and GDS values. Therefore, the nonparametric Spearman correlation coefficient (ρ) was used to evaluate the interdependence between the characteristics (the correlation of stress with age, the GDS, the BMI, and visceral fat). The non-parametric Wilcoxon rank test was used to analyze the pre-post psychological parameters. Due to the sensitive nature of the data analyzed in the current study (protected medical information), they are available from the authors upon request.

The sample size for our study was a minimum of 40 subjects with a confidence level of 95%, a fraction size estimated at 40%, and an assumed maximum error of 15%.

## 3. Results

### 3.1. First Measurement Point

The detailed characteristics of the study group at the beginning of the therapeutic program are shown in Table 1.

In addition to obesity, other factors predisposing the subjects to the occurrence of the MetS were present in the study group. One of these was hypertension, present in 28 women and untreated in only one person. Another factor was the fasting plasma glucose of more than 100 mg/dL or the treated type II diabetes, which occurred in 44% of the study group. A raised triglyceride level was detected in 28% of participants, among whom 67% were under pharmacological treatment. The results are presented in Table 2.

First, the body composition results were analyzed, including the parameter determining the amount of visceral fat tissue, which is an indicator of central obesity (Table 3). The analysis was necessary to assess the risk of metabolic syndrome.

### 3.2. Correlation Analysis

At the beginning of the therapeutic program, stress correlated with the level of depressive symptoms (*p* = 0.00, R = 0.61), the BMI value (*p* = 0.02, R = 0.39), and the amount of visceral fat (*p* = 0.05, R = 0.33). Across the stress components, BMI correlated most strongly with external and intrapsychic stress (*p* = 0.02, R = 0.4), and body fat correlated with internal stress (*p* = 0.03, R = 0.36). This means that the higher the level of perceived stress, the worse the sense of well-being, the higher the BMI value and the amount of visceral fat. This applies to both the result of the general stress assessment and to its components: Emotional tension, external stress, and intrapsychic stress. R-values in the range of 0.2–0.4 are interpreted as low correlation (visible relationship), while 0.4–0.6 corresponds to moderate correlation (significant relationship), and a score of 0.6–0.8 indicates high correlation (significant relationship). Table 4 presents the results of the correlation analysis. The correlation coefficients significant at the level of *p* < 0.05 are indicated by the “*” symbol.

### 3.3. Second Measurement Point: Pre-Post Analysis

The GDS and the PSQ were applied again after 3 months of regular participation in the therapeutic program. The results indicated a significant reduction in stress intensity (overall stress score and external stress score). A comparison of the pre-post scores obtained with the psychological tools is provided in Table 5. The distribution of the PSQ STEN values in the pre-post analysis is shown in Table 6.

The distribution of the PSQ STEN values allows the observation of a noticeable change (*p* < 0.01; Z = 3.9) in the level of perceived stress, especially for the 1 and 2 STEN values (an increase in the number of people with the lowest levels of stress). Moreover, it is worth noting the reduction in the number of people with the highest levels of stress (6–10 STEN) (Table 6).

## 4. Discussion

The purpose of this study was to determine the impact of multimodal therapeutic approach on self-perceived stress in women with metabolic syndrome (MetS), and to analyze the relationship between the level of perceived stress, mood, and the components of the MetS in women aged 60 years and older. The participants of the Mental Health Promotion Program were postmenopausal women. They decided to participate in the project to take care of their health and improve their quality of life. A unique aspect of the study was a group multimodal therapeutic approach of a biopsychosocial nature. Activities offered by the Foundation for Senior Citizen Activation SIWY DYM are aimed at supporting the treatment of civilization diseases through non-pharmacological forms of therapy. The project included activities such as general fitness training, dance, as well as health-promoting education and psychoeducation.

The enrichment of the therapeutic activities with art-based activities, i.e., active participation in dance sessions, may have contributed to the effectiveness of the described project in terms of stress reduction. Obviously, as with all of the multimodal approaches, we are not able to determine the extent to which individual elements of the project contributed to the outcome achieved. The positive impact of this approach comes precisely from the fact that the various components of the project complement each other. In this case, the components were physical exercise, dance, and health education. 

The possibility of effective use of dance not only in the case of physical, but also psychological and social problems, makes it an integral element of the multimodal therapeutic approach in our experiment. Unfortunately, there is not enough research on the subject that would allow the comparison of our findings. One of the few studies examining the application of dance to high-risk MetS is the study on the impact of rumba dance and nutrition education interventions on cardiovascular risk factors in a group of people with MetS in rural Colombia. The researchers reported that the program using rumba and muscular strengthening, combined with nutrition education, favorably modified cardiovascular risk factors in people with MetS [20]. Hofgaard et al. [21] studied the impact of a Faroese chain dance intervention on the health profile, mobility, and postural balance in elderly subjects. They found that the Faroese chain dance has a beneficial effect on postural balance and physical function, and most likely on blood pressure and body fat content, in elderly participants after only 6 weeks of training. 

Interestingly, although the researchers’ focus was on assessing changes related to physical functioning, they also emphasized the importance of dance as an integral part of identity and culture. In addition, the researchers pointed out the social benefits of dance intervention in a specific ethnic group. Dance improves social interaction and enjoyment and may overcome barriers to physical activity in older adults, which was also reflected in our study [21,22]. During our project, the group of participants became close to one another, the women formed bonds, supported each other, overcame the barriers of shame, and decided to freely express their emotions through dance. 

In general, physical exercise affects both physical health parameters and mental health. Moreover, exercise has a positive effect on conditions often co-occurring with mood disorders (e.g., anxiety, pain, and insomnia) [23]. Chronic unresolvable stress leads to the development of mood disorders and cardiovascular disease [24]. In our study, a pre-post comparison showed that the level of perceived stress was significantly reduced after 3 months of regular participation in the therapeutic program. Comparing the results of the cited studies with the results obtained in our project, it can be concluded that the women who participated in the project received appropriate help. These are very promising findings, although they should be treated with caution due to the nature of the study design and the small sample size.

The correlations we explored in this study were largely in line with expectations (the higher the stress level, the worse the mood). However, the correlations of particular stress components (emotional tension, external stress, intrapsychic stress) with the BMI and visceral fat were interesting. The problem of overweight and obesity is related not only to eating habits, but is undeniably associated with mental factors such as the perceived stress or the experienced internal conflicts, including intramental conflicts. Moreover, obesity correlates with the deterioration of executive functions. It is believed that new interventions should be developed, which would take into account mood and emotion regulation in the treatment of obesity [25]. In our opinion, it is worthwhile for future studies to focus on the in-depth meaning and impact of particular stress components on the components of MetS. This could help in better adapting the activities and content of health education to the needs of patients with MetS.

It is worth noting that addressing dietary modification and changing eating habits was an important part of the education in this program. Perhaps the next step could be for a specialist to develop a personalized diet for each participant. Castro-Barquero et al. [26] reported that lifestyle modifications, especially dietary habits, are the main therapeutic strategy for the treatment and management of MetS, but the most effective dietary pattern for its management has not been established. Specific dietary modifications, such as improving the quality of the foods or changing macronutrient distribution, showed beneficial effects on MetS parameters. Moreover, energy-restricted dietary patterns and an increase in physical activity are crucial to improve the metabolic disturbances observed in metabolic syndrome patients. However, in our opinion, these restrictions should be introduced gradually, when the motivation of the participants is already very strong.

The prevalence of the MetS increases after the menopause and its dominant component (obesity) increases the prevalence and severity of menopausal symptoms [27]. Evidence shows that individuals with MetS have a significantly reduced health-related quality of life compared to those without the syndrome. Furthermore, the deterioration of the overall physical and mental health increases along with the number of MetS components presented in a patient [27,28,29]. Results from other studies suggest that the relationship between MetS, obesity, mood, and cardiovascular risk is complex. In fact, women with depression, emotional tension, stress, and anger show a higher risk of developing insulin resistance, MetS, and cardiovascular disease [27,30]. Eschweiler [31] argued that the multifactorial genesis of depression in the elderly includes psychosocial, vascular, and metabolic factors and requires multimodal treatment modules at the biological and psychosocial levels. We consider that MetS should be treated analogously. The analysis of the literature and our previous study [7] show that a biopsychosocial approach may be crucial in the treatment of these complex problems. We see our current study as an exploratory one that will allow us to continue our research and optimize therapeutic interventions in a group of postmenopausal women at high risk of MetS.

Civilization diseases are a major problem all over the world. The complexity of health problems concerning women aged 60 years and older make it necessary to develop effective and low-cost strategies involving biopsychosocial interventions. The incorporation of the general fitness training, dancing, health-promoting education, and psychoeducation sessions as therapeutic procedures can be an effective and interesting way to support the multi-faceted treatment of civilization diseases, including MetS. At the end of the project, all of the participants expressed their willingness to continue their participation in the classes, which is very important as there is a need to conduct long-term health-promoting activities in the age group in question. 

## 5. Limitations

A major limitation of our study is the lack of a control group. Therefore, a randomized clinical trial should be planned for the next stage of the investigation. Both a control group without any intervention and a control group of patients participating in regular physical activity but without therapeutic components should be considered. The strength of the findings will be greater if the group of patients with MetS is significantly larger.

## 6. Conclusions

The level of perceived stress in the study group was related to the presence of depressive symptoms, BMI, and the amount of visceral fat in the study group. A multimodal therapeutic program including general exercise training, dance, health education, and psychoeducation is an interesting approach to reduce stress levels. However, due to the small sample size and other limitations of the project, the results should be treated with caution.

## Figures and Tables

**Table 1:** Participants’ baseline characteristic, *n* = 43.

Total *n* = 43 (mean ± SD)		
Age (years)		68.6 ± 6.5
Mass (kg)		73.2 ± 17.9
Height (m)		159.6 ± 6.0
BMI (kg/cm^2^)		28.9 ± 6.2
Waist Circumference (cm)		97 ± 14.3
Hips Circumference (cm)		113 ± 16.4
WHR		0.86 ± 0.08
Education (%)	Basic/vocational	15
	Secondary	51
	Higher education	34
Marital status (%)	Married	37
	Single	24
	Divorced	7
	Widowed	32
Body weight classification (%)	Normal weight	25
	Overweight	39
	Class I obesity	22
	Class II obesity	14
Mood and well-being (%)	Lack of depression	76
	Moderate depression	22
	Severe depression	2

Abbreviations: BMI—body mass index; WHR—waist-hip ratio; SD—standard deviation.

**Table 2:** Laboratory tests results and blood pressure of the women in the study group.

Feature		Mean	SD	Minimum	Maximum
Blood pressure (mmHg)	systolic	134.9	21.2	90	184
	diastolic	74.8	10.2	52	98
Cholesterol (mg/dL)	total	211.5	42.1	149	342
	HDL	70.6	23.8	42	183
	LDL	118.7	39.9	63	238
Triglycerides (mg/dL)		120.5	47.2	48	253
Blood sugar level (mg%)		98.4	13.8	60	125

Abbreviations: HDL—high-density lipoprotein; LDL—low-density lipoprotein; SD—standard deviation.

**Table 3:** Parameters of the distribution of body composition characteristics of the women examined.

Feature	Mean	SD	Minimum	Maximum
Fat (%)	38.9	6.9	21.1	50.2
Water Composition (%)	44.2	4.6	36.5	55.2
Muscle Mass (kg)	41.7	7.4	28.6	58.9
Bone Mass (kg)	2.2	0.4	1	3.1
Basal Metabolic Rate (kcal)	1332.8	238.3	983	1947
Visceral Fat	11	2.9	6.5	19

**Table 4:** The coefficients of correlation of stress with age, the GDS, the BMI, and visceral fat in the study group.

Stress Components	Spearman’s Rank Correlation Coefficient			
	Age	GDS	BMI	Visceral Fat
Emotional tension	0.1, *p* = 0.52	0.57 * *p* = 0.00	0.31 *p* = 0.06	0.3 *p* = 0.08
External stress	0.13, *p* = 0.40	0.53 * *p* = 0.00	0.4 * *p* = 0.02	0.32 *p* = 0.06
Intrapsychic stress	0.19, *p* = 0.23	0.52 * *p* = 0.00	0.4 * *p* = 0.02	0.36 * *p* = 0.03
General PSQ score	0.15, *p* = 0.35	0.61 * *p* = 0.00	0.39 * *p* = 0.02	0.33 * *p* = 0.05

Abbreviations: GDS—geriatric depression scale; BMI—body mass index; PSQ—perception of stress questionnaire; *—correlation coefficients significant at *p* < 0.05.

**Table 5:** Effects of a 3-month intervention on the results of GDS and PSQ scales in the Wilcoxon rank test.

*N* = 43	Mean	SD	Minimum	Maximum	*p*-Value	Z	T
**GDS**							
Before	6.95	5.66	0	30			
After	6.79	5.18	0	20			
Change	0.16				NS	0.337	246.00
**General PSQ Score**							
Before	60.53	18.28	23	104			
After	49.81	16.41	22	83			
Change	10.72				<0.001	4.095	124.00
**Emotional Tension**							
Before	18.21	6.97	7	32			
After	18.33	7.34	7	35			
Change	−0.12				NS	0.283	351.00
**External Stress**							
Before	16.91	5.32	8	30			
After	14.30	4.70	7	25			
Change	2.61				<0.001	3.734	113.00
**Intrapsychic Stress**							
Before	17.91	5.61	7	30			
After	17.19	6.35	7	31			
Change	0.72				NS	1.584	276.50

Abbreviations: GDS—geriatric depression scale; SD—standard deviation; NS—non significant.

**Table 6:** Distribution of stress level scores in the study group in STEN (the higher the STEN, the higher the stress level).

STEN	PRE%	POST%
1	5	23
2	9	19
3	16	9
4	21	16
5	18	19
6	14	7
7	5	5
8	7	0
9	0	0
10	5	2

## Data Availability

The data presented in this study are available on request from the corresponding author. The data are not publicly available due to privacy restrictions.
